# Complete genome sequences of *Alkalilimnicola ehrlichii* strain SGAR-12 isolated from a pilot-scale haloalkaline biodesulfurization installation and type strain *A. ehrlichii* MLHE^T^

**DOI:** 10.1128/mra.00567-25

**Published:** 2025-09-18

**Authors:** Caroline M. Plugge, Suyash Gupta, Sarahi Garcia Villa, Peer H. A. Timmers, Gerard Muyzer

**Affiliations:** 1 Wetsus, European Centre of Excellence for Sustainable Water Technology 361182, Leeuwarden, the Netherlands; 2 Laboratory of Microbiology, Wageningen University and Research 568404 , Wageningen, the Netherlands; 3 Microbial Systems Ecology, Department of Freshwater and Marine Ecology, Institute for Biodiversity and Ecosystem Dynamics, University of Amsterdam 725872 , Amsterdam, the Netherlands; 4 Department of Environmental Sciences for Sustainability, IE University 367407, Segovia, Spain; University of Southern California, Los Angeles, California, USA

**Keywords:** *Alkalilimnicola*, sulfide, biodesulfurization

## Abstract

The sulfide-oxidizing bacterium *Alkalilimnicola ehrlichii* strain SGAR-12 was isolated from a pilot-scale haloalkaline biodesulfurization (BD) installation, and its genome was sequenced. Additionally, the genome of the type strain of the species, strain MLHE^T^, was sequenced. In-depth genomic analysis may reveal differences in the sulfur utilization spectrum of both strains.

## ANNOUNCEMENT

Only two *Alkalilimnicola* species have been isolated: *Alkalilimnicola halodurans* (1) and *A. ehrlichii* (2). *Alkalilimnicola* sp. was recently identified as a dominant player in a haloalkaline BD installation, converting sulfide to sulfur (3–6). We isolated the most dominant sulfide-oxidizing bacterium from a pilot-scale haloalkaline BD system located at Wageningen University Campus, the Netherlands. *Alkalilimnicola ehrlichii* SGAR-12 was enriched in serial dilution under oxygen-limiting conditions (12% v/v) in a haloalkaline mineral medium at pH 8.5 in serum bottles incubated at 35°C. The medium was prepared as described previously (7), i.e., with 20 mM sodium sulfide (Na_2_S•9 H_2_O) and without urea. The highest dilution with growth was streak plated on the same medium (7) with 2% (w/v) washed agar. SGAR-12 was obtained in pure culture and its genome sequenced. Additionally, we sequenced the genome of the type strain *A. ehrlichii* MLHE^T^ (DSM 17681^T^) to facilitate comparative genome analysis.

SGAR-12 was grown in serum bottles under oxygen-limiting conditions (12% v/v) as described above. MLHE-1^T^, from our culture collection, was cultured on medium 1457 as recommended by the DSMZ (https://www.dsmz.de/collection/catalogue/details/culture/DSM-17681).

The genomic DNA from both strains was extracted using the modified CTAB method (8, 9). The same extract was used for sequencing on both the Illumina NovaSeq 6000 and Oxford Nanopore platforms. Unless otherwise stated, default settings were applied for all analyzes.

For Illumina sequencing, the DNA concentration, purity, and integrity were analyzed using the Agilent 5400 fragment analyzer. Genomic DNA was randomly sheared into short fragments, which were then repaired, A-tailed, and ligated with Illumina adapters. Adapter-containing fragments were amplified by PCR, size-selected for ~350 bp, and purified using the Illumina Sequencing Analysis Viewer. Libraries were prepared using the NovaSeq 6000 SP Reagent Kit for the NovaSeq 6000 SP flow cell, producing 150-bp paired-end libraries. The libraries were checked using Qubit and real-time qPCR and then pooled. DNA quality checks, library preparation, and sequencing were carried out at Novogene Limited, Cambridge, UK. Raw read quality was assessed with the Illumina Sequencing Analysis Viewer. The raw data were cleaned for adapter sequences, reads with *N* > 10%, and low-quality reads (Q-score ≤ 5).

For Oxford Nanopore sequencing, the DNA library was prepared using the Nanopore Kit SQK-LSK109 (Oxford Nanopore Technologies, UK) without DNA shearing. To enrich DNA fragments larger than 3 kb, Long Fragment Buffer was used during the washing step before final elution. Sequencing was performed on the MinION platform using a FLO-FLG001 flow cell with 9.4.1 chemistry, followed by base calling with the MinKNOW v21.02.2 and Guppy v4.3.4 protocol. Raw read quality control was conducted using MinKNOW. Hybrid genome assembly was performed using Unicycler v0.4.9 under standard conditions for both strains (10). The quality of the assembled genomes was assessed with CheckM v1.4.0 (11). The assembly generated single circular contigs for both strains. The complete assembly size of SGAR12 and MLHE^T^ was 3,194,001 bp and 3,264,331 bp, respectively. Subsequently, the assembled genomes were annotated using the NCBI with the NCBI Prokaryotic Genome Annotation Pipeline (12). Further genome characteristics are in Table 1.

**TABLE 1:** Genome statistics of *Alkalilimnicola erhlichii* SGAR-12 and MLHE^T^

	Strain SGAR-12	Strain MLHE^T^
BioSample accession no	SAMN472143149	SAMN47214681
No. of Illumina raw reads	8,552,010	9,613,662
No. of Nanopore raw reads	33,508	123,456
Genome size (bp)	3,194,001	3,264,331
Genome coverage Illumina (×)	2.6	2.9
Genome coverage Nanopore (×)	1.2	4.3
Number of contigs	1 (circular)	1 (circular)
N_50_ value Nanopore (Kb)	21.13	20.89
CheckM completeness (%)	99.65	100
CheckM contamination (%)	1.12	0.59
G + C content (%)	67.12	67.54
Total genes	2,924	2,954
RNA genes	58	58
rRNA operons (5S, 16S, 23S)	2, 2, 2	2, 2, 2
Protein-coding genes	2,816	2,883
Pseudo genes	50	13
Genome accession no.	CP184336	CP183314

## Data Availability

Draft genome sequences were deposited at DDBJ/ENA/GenBank via BioProject PRJNA1231520 and PRJNA1231541 for SGAR-12 and *A. ehrlichii*, respectively. All sequence reads are available from NCBI Sequence Read Archive under SRP570540 and SRP570655, for SGAR-12 and MLHE^T^, respectively.
